# Anti-Inflammatory Effects of Concentrated Ethanol Extracts of Edelweiss (*Leontopodium alpinum* Cass.) Callus Cultures towards Human Keratinocytes and Endothelial Cells

**DOI:** 10.1155/2012/498373

**Published:** 2012-10-10

**Authors:** Lulli Daniela, Potapovich Alla, Riccardo Maurelli, Dellambra Elena, Pressi Giovanna, Kostyuk Vladimir, Dal Toso Roberto, De Luca Chiara, Pastore Saveria, Korkina Liudmila

**Affiliations:** ^1^Laboratory of Tissue Engineering and Skin Pathophysiology, Dermatology Institute (IDI IRCCS), Via Monti di Creta 104, 00167 Rome, Italy; ^2^Biology Department, Belarus State University, Nezavisimosti Avenue 4, 220050 Minsk, Belarus; ^3^Institute of Biotechnology Research (I.R.B. S.r.l.), Via Lago di Tovel 7, 36077 Altavilla Vicentina, Italy

## Abstract

Edelweiss (*Leontopodium alpinum* Cass.) is traditionally employed in folk medicine as an anti-inflammatory remedy. In nature, the plant is sparsely available and protected; therefore production of callus cultures was established. A concentrated ethanolic extract of culture homogenate, with leontopodic acid representing 55 ± 2% of the total phenolic fraction (ECC55), was characterized for anti-inflammatory properties in primary human keratinocytes (PHKs) and endotheliocytes (HUVECs). Inflammatory responses were induced by UVA+UVB, lipopolysaccharide (LPS), oxidized low-density lipoprotein (oxLDL), and a mixture of proinflammatory cytokines. Trichostatin A, a sirtuin inhibitor, was used to induce keratinocyte inflammatory senescence. ECC55 (10–50 **μ**g/mL) protected PHK from solar UV-driven damage, by enhancing early intracellular levels of nitric oxide, although not affecting UV-induced expression of inflammatory genes. Comparison of the dose-dependent inhibition of chemokine (IL-8, IP-10, MCP-1) and growth factor (GM-CSF) release from PHK activated by TNF**α** + IFN**γ** showed that leontopodic acid was mainly responsible for the inhibitory effects of ECC55. Sirtuin-inhibited cell cycle, proliferation, and apoptosis markers were restored by ECC55. The extract inhibited LPS-induced IL-6 and VCAM1 genes in HUVEC, as well as oxLDL-induced selective VCAM1 overexpression. *Conclusion.* Edelweiss cell cultures could be a valuable source of anti-inflammatory substances potentially applicable for chronic inflammatory skin diseases and bacterial and atherogenic inflammation.

## 1. Introduction

Since ancient time, plants have been undiminished sources of products traditionally used for medicinal and skin care purposes. In our highly technological era, natural substances of plant origin remain major active principles of numerous drugs and “ceuticals” (nutraceuticals and cosmeceuticals). According to pharmacological statistics, 12 out of 40 anti-inflammatory drugs approved between 1983 and 1994 worldwide were derived from or based on secondary plant metabolites, mainly polyphenols [1]. Recently, a number of *in vitro* and *in vivo* animal studies have provided first scientific evidence for ethnopharmacological use of *Leontopodium alpinum *Cass. (family Asteraceae, named also Edelweiss) as a potent anti-inflammatory [2–5] and antibacterial remedy [6, 7]. Edelweiss is a famous plant sparsely distributed in high mountains of Europe and Asia at altitude of 1800–3000 m. The rare plant spontaneously grows in inaccessible areas, and it is protected in many countries [8]. In European folk medicine, aerial parts (leaves, flowers, and stems) of Edelweiss have been used to attenuate inflammation and pain in respiratory system (tonsillitis, bronchitis, and pneulmonitis), gastrointestinal system (gastritis and colitis) [8], joints (rheumatoid arthritis) [9], and tumor-affected tissues (skin and connective tissues over breast cancer) [10]. In laboratory experiments, extracts of dried roots of Edelweiss possessed anti-inflammatory properties [2, 5]. During the last few years, anti-inflammatory properties of Edelweiss have been extensively exploited in cosmetic preparations in order to possibly protect the skin against solar irradiation-induced chronic skin inflammation, eventually leading to premature aging and high incidence of skin tumors [10]. 

The great majority of health properties of this plant have been attributed to the presence and the spectrum of polyphenolic secondary metabolites. Edelweiss contains a wide variety of polyphenols belonging to classes of phenylpropanoids (phenolic acids, glycosides, flavonoids, coumarines, and lignans), terpenes (sesquiterpenes and diterpene acids), and alkaloids (benzofuran and pyrane derivatives) [11]. The ethnopharmacological use of Edelweiss is supported by the isolation of a number of phenylpropanoids possessing anti-inflammatory and antibacterial properties from the plant root, its aerial parts, or from hairy root cultures [2]. The analytical assessment of extracts of aerial parts of Edelweiss revealed glycosides and aglycones of flavonoids (luteolin, quercetin, and apigenin) as well as high content of leontopodic, chlorogenic, and 3, 5-dicaffeoylquinic acids [12]. By chemical structure, leontopodic acids (isomers A and B, LA1, and LA2, resp.) contain four phenylpropanoid moieties connected to a backbone of glucaric acid. Later publications described LA1 and LA2 as having the greatest impact on beneficial biological effects of Edelweiss herb extracts [6, 11]. The extracts also display antioxidant, DNA-protecting, and analgesic effects, proven in different *in vivo* models [5]. 

Taking into account growing public concern about overexploitation of rare plant species for medicinal and cosmetic use, plantations of cultivated Edelweiss were established in Switzerland [10]. Recent advances in molecular biology and biochemistry of secondary plant metabolites, metabolic bioengineering, and scaleup production of desired molecules in stable plant cell/tissue cultures seem to open a new era for classical ethnopharmacologically relevant plants [13]. Early studies of aseptic plant cultures *in vitro* reviewed by Thorpe [14] led to the development of hairy root, embryo, and callus/tissue cultures. Plant cell/tissue cultures subjected to proper eliciting seem to be an ideal “chemical factory” for the biotechnological production of secondary metabolites providing controlled, uncontaminated, and all-year-round biosynthesis of complex molecules. Moreover, plant polyphenols synthesized in plant cell cultures are not subjected to polymerization process, which usually takes place in plants to construct cell walls and to close wounds [15]. Hence, the yield of monomeric polyphenolic molecules could be much higher in cultures than in mature plant parts, and their extraction and purification are greatly simplified.

In the present work, mechanisms of anti-inflammatory activity of concentrated ethanol extracts of *Leontopodium alpinum* callus cultures, containing phenolic fraction with 55% of LA1 and LA2, were evaluated in two *in vitro* models of primary human keratinocytes and endothelial cells activated by physiologically relevant proinflammatory stimuli, such as solar UV irradiation, bacterial lipopolysaccharide (LPS), oxidized low-density lipoprotein (oxLDL), mixture of proinflammatory cytokines (tumor necrosis factor alpha + interferon gamma), and sirtuin inhibitor (trichostatin A). Comparison with commercially available pure LA showed a prominent anti-inflammatory action of the extract exerted through several distinct molecular pathways that largely depended on the presence of LAs.

## 2. Materials and Methods

### 2.1. Solvents and Reagents

All solvents and reagents were of the highest purity available from Sigma Aldrich, Milan, Italy, if not indicated otherwise. As reference plant polyphenols, commercial LA, quercetin (Qr), and resveratrol (Rv) were used.

### 2.2. Plant Cell Cultures

In order to protect rare species of Edelweiss from extinguishing and facilitate synthesis and extraction of biologically active substances, stable callus cultures derived from *Leontopodium alpinum* seeds were established. The growth and specific eliciting conditions allowed achieving a higher yield of LAs and chlorogenic acids compared with extracts from the mature plant parts. 

Cell cultures of *Leontopodium alpinum* (Edelweiss, ECC) were obtained from commercially available seeds. The seeds were first sterilized and then laid on a Petri dish until small sterile plantlets were grown. Then, explants were collected and transferred to a new solid agar plate with Gamborg B5 medium (Sigma Aldrich, Milan, Italy) containing 20 g/L sucrose, 1 g/L plant peptone, 1 mg/L kinetin, 1 mg/L naphthalene acetic acid, and 0.2 mg/L indol acetic acid at pH 6.5. Calli grown on solid Gamborg B5 medium were subjected to subculture for at least 3 months and subsequently were used to inoculate Erlenmeyer flasks of 1 L volume, with the final liquid Gamborg B5 medium content of 250 mL for each flask. After 14 days of fermentation at 25°C in the dark, the cells were collected, and the secondary metabolites were extracted.

### 2.3. Plan Cell Extract Preparation and Analysis

Fifteen-day-old cell cultures (total volume 15 L) were filtered, and the medium was discarded. After addition of 15 g of solid ascorbic acid, cells were extracted with two volumes of ethanol, homogenized, centrifuged, and concentrated under reduced pressure (ECC). The phenolic compounds present in the aqueous residue were quantitatively recovered by solid-phase extraction using a column (7 × 50 cm) containing 1.5 kg of XAD-4 resin suspended in 5% aqueous HCOOH. The column was washed with H_2_O and eluted with 80% ethanol. Fractions containing caffeic derivatives were concentrated under reduced pressure, and the aqueous residue freeze dried, yielding 14 g of grey powder. 

The detailed analysis of the callus cultures extract was performed by HPLC-ESI-MS using an HPL chromatograph (System Gold 127, Beckman Coulter) connected to a mass-spectrometer (Esquire 6000) equipped with an electrospray ionization and ion-trapping system (ESI, Bruker Daltonics). All analyses were repeated twice. All reagents and reference molecules of phenolics used were of analytical grade (Sigma Aldrich, Milan, Italy), and HPLC solvents were of gradient grade. 

In order to separate the different caffeic acid derivatives, the powder was resuspended in H_2_O and completely dissolved with a few drops of concentrated NH_4_OH. Solution was submitted to Sephadex LH20 CC (8 × 24 cm) suspended in aqueous 15% ethanol containing 1% HCOOH. The column was first eluted with the same solvent, and fractions (100 mL each) were collected and analyzed by HPLC. Fractions 44–52 were pooled and lyophilized yielding 2.75 g of pure chlorogenic acid. Elution was continued with aqueous 30% ethanol + 1% HCOOH, and fractions 75–93 gave 6.53 g of an LA1 and LA2 mixture. In order to separate leontopodic acids, freeze-dried fractions 75–93 were submitted to a second chromatography in reverse phase on RP-C18 LiChrosphere (Merck, Darmstadt, Germany). A column (cm  5 × 40), filled with resin suspended in aqueous CH_3_CN 1% added with HCOOH 1%, was used. The column was eluted with increasing amounts of  CH_3_CN added of 1% HCOOH, and 150 mL fractions were collected. Pure LA1 and LA2 were recovered in the fractions with 20% of  CH_3_CN and 50%, respectively. In order to separate LA1 and LA2, the powder obtained by freeze drying of fractions 75–93 was submitted to a second-column chromatography in reverse phase on RP-C18 LiChrosphere (Merck, Darmstadt, Germany) using a column (cm  5 × 40), filled with resin suspended in aqueous 1%  CH_3_CN + 1% HCOOH. The column was eluted with increasing amounts of  CH_3_CN together with 1% HCOOH, and 150 mL fractions were collected [16]. The material (ECC55), which was used in biological experiments, was finally analysed by HPLC-ESI-MS method.

### 2.4. Human Cell Cultures

#### 2.4.1. Primary Human Keratinocytes (PHKs)

PHKs obtained from skin biopsies of healthy subjects (*n* = 5) [17] were seeded onto 9.6 cm^2^ wells (60,000 cells/well) and cultured in a 5% CO_2_ humidified atmosphere in keratinocyte growth medium: DMEM and Ham's F12 (both from Lonza, Visp, Switzerland) media (2 : 1 mixture) containing 10% fetal bovine serum (Invitrogen, USA), 0.18 mM adenine, 0.4 *μ*g/mL hydrocortisone (both from Calbiochem, USA), 5 *μ*g/mL insulin, 0.1 nM cholera toxin, 2 nM triiodothyronine, 50 IU to 50 *μ*g/mL penicillin-streptomycin, 4 mM glutamine, and 10 ng/mL epidermal growth factor (Austral Biologicals, USA). The protocol of the study was approved by the local Ethical Committee (IDI IRCCS—San Carlo Hospital), and informed consent for skin biopsy was signed by all the participants.

#### 2.4.2. Human Endothelial Cells

Human umbilical vein endothelial cells (HUVECs) were purchased from Lonza (Verviers, Belgium) and were cultured in endothelial cell growth medium (EGM-2 BulletKit, Lonza, Verviers, Belgium) and supplemented with 2% fetal bovine serum (FBS), at 37°C in humidified atmosphere containing 5% CO_2_. All experiments were performed with HUVEC cells at the fourth to seventh passage.

### 2.5. Induction of Inflammatory Responses

Inflammatory response to low-dose UVA+UVB irradiation (30 s, UVA 1.0 J/cm^2^ + UVB 0.1 J/cm^2^) produced by solar simulator (Dermalight Vario with filter A2, Dr. Hoehnle AG, UV Technology, Planegg, Germany) with emission spectrum from 280 nm and emission peak at 375 nm was evaluated. The light effluence rate on the cell monolayer was 40 mW/cm^2^. ECC55 (25 *μ*g/mL) or reference plant polyphenols (25 *μ*g/mL) or corresponding vehicle was added 30 min before UV exposure [18]. 

Two days after seeding, PHKs were treated with different doses (1 to 100 *μ*g/mL) of ECC extracts for 48 hours. Then, 100 nM Trichostatin A (TSA) was added, and 24 h later the medium was changed for starvation medium: DMEM, glutamine (4 mM), and penicillin-streptomycin (50 IU and 50 *μ*g/mL, resp.). PHKs were cultivated for 24 h and then collected and used for further analysis.

PHKs were preincubated with escalating concentration of either ECC55 or commercial LA (from 1 to 100 *μ*g/mL) for 1 h. Then, 100 ng/mL of tumor necrosis factor alpha (TNF*α*) and 100 U/mL interferon gamma (IFN*γ*) were added. The combination of two proinflammatory factors induced maximal expression and release of cytokines and chemokines from PHK [19]. After 24 h, concentrations of interleukin 8 (IL-8), interferon gamma-produced protein of 10 kDa (IP-10), granulocyte macrophage colony-stimulating factor (GM-CSF), and monocyte chemotactic protein 1 (MCP-1) were measured by enzyme-linked immunosorbent assay (ELISA).

HUVEC cells were treated with LPS (055 : B5 Sigma-Aldrich, 1 *μ*g/mL) or vehicle (water) in the presence or absence of ECC55 or reference plant polyphenols (25 *μ*g/mL) for 3-4 h. Then, cells were scraped, and the whole cell homogenates were prepared for further extraction of RNA as described below.

Oxidised low-density lipoproteins (oxLDLs) were prepared as described previously [20]. In brief, human native LDL was diluted to a 200 *μ*g protein/mL in phosphate-buffered saline (pH 7.4) and incubated with 15 *μ*g/mL human myeloperoxidase (Calbiochem, San Diego, CA, USA), 50 *μ*M nitrite, and 0.6 mM hydrogen peroxide at 37°C for 30 min. To stop the reaction and to remove the excess of hydrogen peroxide, 0.1 mg/mL of catalase was added. Lipid peroxides were measured using ProxiDetect Kit from Sigma Aldrich (Milan, Italy). Their content in oxLDL ranged from 190 to 210 mol/mol LDL. In the *in vitro* experiments, 10 *μ*g protein/mL oxLDL dissolved in DMSO was added to HUVEC cultures. Cells were incubated with oxLDL in the presence or absence of ECC55 for 1 h or 4 h. The control cultures were incubated with vehicles for ECC55 (ethanol) and/or oxLDL (DMSO) added at the same concentrations (0.2% v/v) as in the experimental cultures.

### 2.6. Assessment of Inflammatory Responses

#### 2.6.1. Total RNA Isolation and RT-PCR

Total RNA was isolated from frozen HUVEC and PHK using the GenElute Mammalian Total RNA Kit in accordance with manufacturer's instructions. Total RNA (3 *μ*g) was reverse transcribed using the iScript cDNA Synthesis Kit at 25°C for 5 min and 42°C for 30 min, followed by 85°C for 5 min in a final reaction volume of 60 *μ*l. cDNA was amplified with IQ SYBR green Supermix using the MiniOpticon real-time PCR detection system (all reagents were from Bio-Rad, Hercules, USA). Melt curve analysis was performed to confirm the specificity of the amplified products. Signal of housekeeping gene beta-actin was used as the reference. Changes were calculated with the comparative Ct method (ΔΔCt) according to [21]. Total RNA from each experimental point was reverse-transcribed in duplicate, and all PCR amplifications were repeated two times. The following primer sets were designed using Primer-BLAST (NCBI) and were synthesized by Eurofins MWG Operon (Ebersberg, Germany): *β*-actin fwd: 5′-AATCTGGCACCACACCTTCTAC-3′; *β*-actin rev: 5′-ATAGCACAGCCTGGATAGCAAC-3′; 18S rRNA fwd: 5′-TCCCCCAACTTCTTAGAGG-3′; 18S rRNA rev: 5′-GCTTATGACCCGCACTTAC-3′; iOS fwd: 5′-TACTCCACCAACAATGGCAA-3′; iOS rev: 5′-ATAGCGGATGAGCTGAGCAT-3′; IL-6 fwd: 5′-GTGTGAAAGCAGCAAAGAG-3′; IL-6rev: 5′-CTCCAAAAGACCAGTGATG-3′; TNF-*α* fwd: 5′-TCCTTCAGACACCCTCAACC-3′; TNF-*α* rev: 5′-AGGCCCCAGTTTGAATTCTT-3′; IL-1*α* fwd: 5′-TGGCTCATTTTCCCTCAAAAGTTG-3′; IL-1*α* rev: 5′-AGAAATCGTGAAATCCGAAGTCAAG-3′; COX-2 frw: 5′-TTCTCCTTGAAAGGACTTATGGGTAA-3′; COX-2 rev: 5′-AGAACTTGCATTGATGGTGACTGTTT-3′; SOD-2 frw: 5′-GGCCTACGTGAACAACCTGAA-3′; SOD-2 rev: 5′-CTGTAACATCTCCCTTGGCCA-3′; MCP 1frw: 5′-AAGCAGAAGTGGGTTCAGGA-3′; MCP1 rev: 5′-TAAAACAGGGTGTCTGGGGA-3′; MMP 1frw: 5′-TGTGGACCATGCCATTGAGA-3′; MMP 1rev: 5′-TCTGCTTGACCCTCAGAGACC-3′; MMP 9frw: 5′-GGAGACCTGAGAACCAATC-3′; MMP9 rev: 5′-CACCCGAGTGTAACCATAG-3′; IL-8 frw: 5′-GTCCTTGTTCCACTGTGCCT-3′; IL-8 rev: 5′-GCTTCCACATGTCCTCACAA-3′; ICAM 1frw: 5′-CCCATGAAACCGAACACAC-3′; ICAM 1rev: 5′-ACTCTGTTCAGTGTGGCACC-3′; VCAM fwr: 5′-TCTCATTGACTTGCAGCACC-3′; VCAM rev: 5′-CTCATTCGTCACCTTCCCAT-3′; IFN*γ* frw: 5′-GCGAAAAAGGAGTCAGATGC-3′; IFN*γ* rev: 5′-CAGGACAACCATTACTGGGA-3′; IP-10 frw: 5′-GGGAGCAAAATCGATGCAGTGCT-3′; IP-10 rev: 5′-GCAGCCTCTGTGTGGTCCATCC-3′; NOX1 frw: 5′-TTAACAGCACGCTGATCCTG-3′; NOX1 rev: 5′-TGTGGAAGGTGAGGTTGTGA-3′.

Both *β*-actin and 18 S rRNA were used as housekeeping genes and served for normalization of all the other genes expression.

#### 2.6.2. ELISA for Cytokine Expression

The levels of IL-8, IP-10, GM-CSF, and MCP-1 in the conditioned medium of PHK stimulated by the mixture of TNF*α* and IFN*γ* were measured after 24 h by using OptELA enzyme-linked immunosorbent assay kits from R&D, USA, as previously described [17].

#### 2.6.3. Protein Extraction and Western Blotting

For immunoblots, PHKs were extracted on ice by lysis with radio immunoprecipitation assay (RIPA) buffer. Equal amounts of samples (50 *μ*g) were subjected to electrophoresis on 12.5% SDS-polyacrylamide gels and transferred to polyvinylidene difluoride (PVDF) filters (Immobilon-P; Millipore). Filters were soaked in 5% nonfat dry milk/TBS (20 mM Tris-HCl, pH 7.5, and 500 mM NaCl) at 4°C overnight. Western blot was performed using the following monoclonal or polyclonal antibodies: anti-p16^INK4a^, anti-p53, anti-p63, anti-PCNA, anti-GAPDH (all from Santa Cruz Biotechnology, Inc., CA, USA), anti-p14^ARF^ from Oncogene (Cambridge, MA, USA), and anti-p21^Waf1^ (a kind gift from Kristian Helin, University of Copenhagen, Denmark). Filters were incubated for 2 h at room temperature, washed three times with a solution containing 20 mM Tris-HCl, pH 7.5, 500 mM NaCl, and 0.05% Tween-20, and finally incubated for 1 h with sheep anti-mouse horse radish peroxidase-labeled immunoglobulin diluted at 1 : 5000 (GE Healthcare, UK).

#### 2.6.4. Assay for Intracellular NO Levels

PHKs were grown to subconfluence in 24-well culture plates, and intracellular NO was detected using 4, 5-diaminofluorescein diacetate (DAF-2DA) [22]. In brief, cells were loaded with 2.5 *μ*M DAF-2DA for 60 min at 37°C. After staining, keratinocytes were washed and fixed by 2% paraformaldehyde for microscopic examination. To quantify the data, cells were detached from culture plates, and the fluorescence of suspended cells was measured on a Shimadzu RF-5301 spectrofluorimeter (*λ*_ex_ = 488 nm  and  *λ*_fl_ = 530 nm). 

#### 2.6.5. Assessment of Cell Growth Rate, Vitality, and Membrane Integrity

Effects of proinflammatory stimuli and their combination with ECC55 or reference plant polyphenols on keratinocyte growth and viability as well as membrane integrity were assessed after 24 h by standard assays. To evaluate the keratinocyte proliferation, subconfluent cultures were trypsinized, and cells were counted under microscope. Cell survival was determined by (3-4, 5-dimethylthiazol-2yl]-diphenyl tetrazolium bromide (MTT) colorimetric assay as previously described [23]. Results are the mean values of 12 independent measurements.

### 2.7. Statistics

Statistical evaluation was carried out with the corresponding software package for Windows XP. The results of all experiments are expressed as the mean values ± S.D. To assess the difference between experimental groups, Student's *t*-test for unpaired data was applied, and differences were considered to be significant at *P* < 0.05.

## 3. Results and Discussion

### 3.1. Analysis of *Leontopodium alpinum* Callus Cell Cultures and Their Extract Enriched in Leontopodic Acids (ECC55)

Results of HPLS-MS-ESI analysis of ethanolic extracts of callus cell cultures are present in Table 1. They are confronted with the previously published analytical determinations of similar fractions of aerial parts of Edelweiss plant [12]. It could be noticed that, unlikely plant parts, ECC does not contain flavonoids such as isoquercetin, luteolin, apigenin, and their glycosylated derivatives. However, it does contain approximately 3% of pentahydroxyflavone used for the plant culture growth. The concentrated extract used in biological experiments (ECC55) contained 41% of LA1, 24% of chlorogenic acid, 21% of 3,5-O-dicaffeoylquinic acid, and 14% of LA2 (Table 1).

### 3.2. Extract of *Leontopodium alpinum* Callus Cell Cultures Enriched in Leontopodic Acids (ECC55) Protects Primary Human Keratinocytes from UVA+UVB-Induced Damage; This Effect Associates to Enhanced Intracellular NO Levels in Irradiated Cells

**Figure 1:**
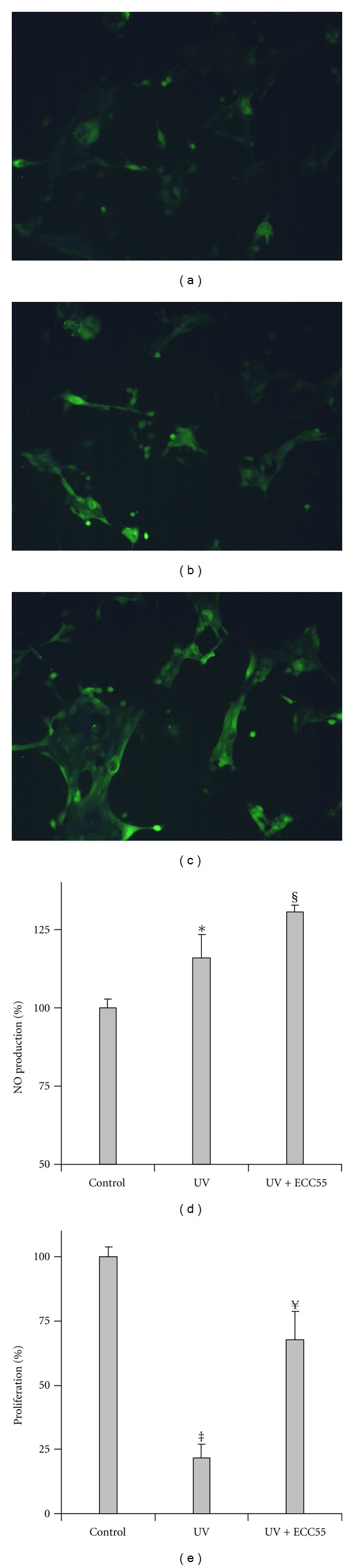
Effects of concentrated ethanol extract of Edelweiss callus cultures (ECC55) on UV-induced keratinocyte damage and intracellular nitric oxide (NO) production. Fluorescent microphotographs of the control untreated keratinocytes (a), keratinocytes 1 h after irradiation by solar simulating UVA+UVB (b), and keratinocytes pretreated with 25 *μ*g/mL of ECC55 for 30 min and then subjected to UVA+UVB exposure (c). Quantification of intracellular fluorescence of NO-specific fluorescent probe DFA-DA (d). Keratinocyte vitality assessed by MTT test. Error bars in all figures correspond to S.D. *P < 0.05 versus sham-irradiated control; ^§^ *P* < 0.05 versus UV irradiation without ECC55; ^‡^ *P* < 0.01 versus sham-irradiated control; ^¥^ *P* < 0.01 versus UV irradiation without ECC55.

Preincubation of PHK with 25 *μ*g/mL ECC55 for 30 min prevented the decrease of cell proliferation observed 1 h after irradiation with solar simulated UVA+UVB (Figures 1(a)-1(c), and 1(e)). At the same time, intracellular level of NO, significantly increased at 1 h after irradiation, was further augmented upon exposure to a combination of ECC55 and UVA+UVB (Figure 1(d)). Plant polyphenols have been shown to protect cells against UVA, UVB, or UVC-irradiation-induced cell death or reversible damage although by different mechanisms [23–25]. The protection could be due to the combination of at least three distinct effects: UV absorption, free radical scavenging + metal chelating = antioxidant, and free radical-independent mechanisms of cell death/prosurvival [26]. Higher plants respond to a variety of oxidative stimuli, including enhanced UV irradiation by increasing the biosynthesis of UVA and UVB-absorbing compounds with pronounced antioxidant properties [15]. LA has been shown to possess remarkable antioxidant properties [5, 11]. An increase in the intracellular levels of NO in UV-exposed keratinocytes occurs by two ways: by the activation of constitutive NO synthases (cNOS) in the early postirradiation period and by the induction of inducible NO synthase (iNOS) at more than 6 h after irradiation [27]. Our measurements done 1 h after irradiation also did not reveal any iNOS gene induction by UV (data not shown); however, NO presence in PHK was slight but statistically significantly increased (Figures 1(b) and 1(c)). Usually, NO produced by cNOS is regarded as a protective reaction to UVB irradiation, and low levels of intracellular NO play an essential role of signal transduction to mount adaptive cellular response [28]. The only risk is connected with the possibility of reaction between NO and superoxide anion-radical ( O_2_^−∙^), with the subsequent generation of the cytotoxic peroxynitrite ONOO^−^ [29]. Distinct membrane-penetrable plant polyphenols in ECC55, such as LA and chlorogenic acids, might scavenge  O_2_^−∙^, thus inducing a shift of NO/ONOO^−^ ratio in favor of cytoprotective and redox signaling NO. Collectively, it seems that phenolic extract of Edelweiss callus cultures could provide a novel mode of skin photoprotection enhancing intracellular production of good nitric oxide while decreasing intracellular levels of bad  O_2_^−∙^.

### 3.3. Extract of *Leontopodium alpinum* Callus Cell Cultures ECC55 Does Not Affect Expression of Inflammatory Genes Induced by UVA+UVB

**Figure 2:**
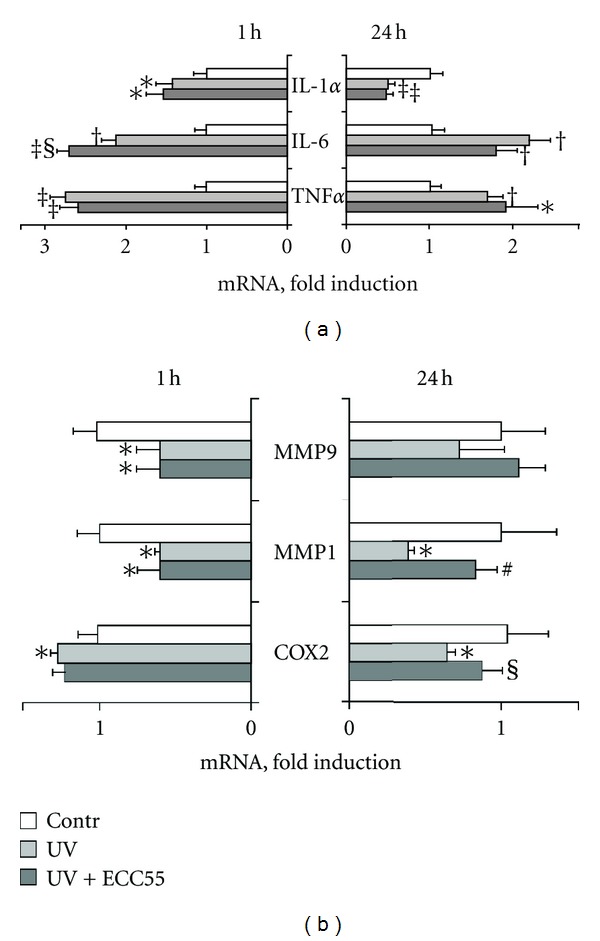
Effects of concentrated ethanol extract of Edelweiss callus cultures (ECC55) on UV-induced gene expression in cultured human keratinocytes. (a) Expression of inflammatory cytokine genes at 1 h and 24 h after irradiation with or without preincubation with ECC55 (25 *μ*g/mL). (b) Expression of matrix metalloproteinases 9 and 1 genes (MMP9 and MMP1) as well as cyclooxygenase-2 gene (COX-2) at 1 h and 24 h after irradiation. Error bars in all figures correspond to S.D. *P < 0.05; ^†^ *P* < 0.01; ^‡^ *P* < 0.001 versus control cells; ^§^ *P* < 0.05; ^#^ *P* < 0.01 versus UV-irradiated cells.

Keratinocytes appear to be not only primary sensors of exogenous and endogenous stresses, such as toxins, microbes, proinflammatory cytokines, and UV, but also key players of the complex stress-inflammation response in the skin [19]. Exposure to moderate physiological doses of UVA+UVB solar-simulated irradiation resulted in the increased or decreased expression of distinct genes seen already at 1 h after irradiation and detected for at least 24 h (Figure 2). Namely, TNF*α* and IL-6, considered as markers of UV-induced inflammatory response in keratinocytes [18], were significantly and durably induced by UV. The preincubation of PHK with 25 *μ*g/mL of ECC55 did not affect gene expression. Fast and transient increase of IL-1a and COX-2 at 1 h after irradiation was also insensitive to ECC55. Suppressive and persistent action of solar UV towards the transcripts of two matrix metalloproteinases 1 and 9 (MMP1 and MMP9) was not changed in the presence of ECC55 (Figure 2). Our data suggest that anti-inflammatory effects of ECC55 at the transcription level could be cell and stimuli dependent. We concluded that adaptive inflammatory responses of normal keratinocytes to low physiological levels of solar irradiation aiming at protection of cellular vitality and restoration of skin cell functions are not disturbed by the Edelweiss cell extract.

### 3.4. Extract of *Leontopodium alpinum* Callus Cell Cultures (ECC55) and Leontopodic Acid Similarly and Dose Dependently Inhibit TNF-Alpha Plus IFN-Gamma-Induced Chemokine (IL-8, MCP-1, and IP-10) and Growth Factor (GM-CSF) Release from Primary Human Keratinocytes

In the course of chronic inflammatory skin disorders, such as psoriasis and atopic dermatitis, epidermal keratinocytes respond to the potent leukocyte-released proinflammatory cytokines tumor necrosis factor (TNF) *α* and interferon (IFN) *γ* by entering a program of enhanced and durable expression of numerous inflammatory mediators. These keratinocyte-derived chemokines and growth factors attract inflammatory cells (granulocytes and monocytes) to the skin and induce their proliferation, thus maintaining the vicious cycle of chronic skin inflammation [17]. We imitated the situation of chronic skin inflammation by adding the combination of TNF*α* + IFN*γ* (T/I) known to induce maximal expression of proinflammatory chemokines and growth factors, to cultivated human keratinocytes. Of note, background release of IL-8 was 0.29 ± 0.04 ng/10^6^ cells, IP-10 was 0.001 ± 0.000 ng/10^6^ cells, GM-CSF was 0.07 ± 0.01 ng/10^6^ cells, and MCP-1 was 0.18 ± 0.02 ng/10^6^ cells. Spontaneous production of IP-10 and MCP-1 was not changed in the presence of either ECC55 or pure commercial LA, while basal GM-CSF and IL-8 levels were decreased by ECC55 and LA dose dependently (data not shown). On these grounds, we concluded that the extract and LA affected intrinsic mechanisms of IL-8 and GM-CSF production.

**Figure 3:**
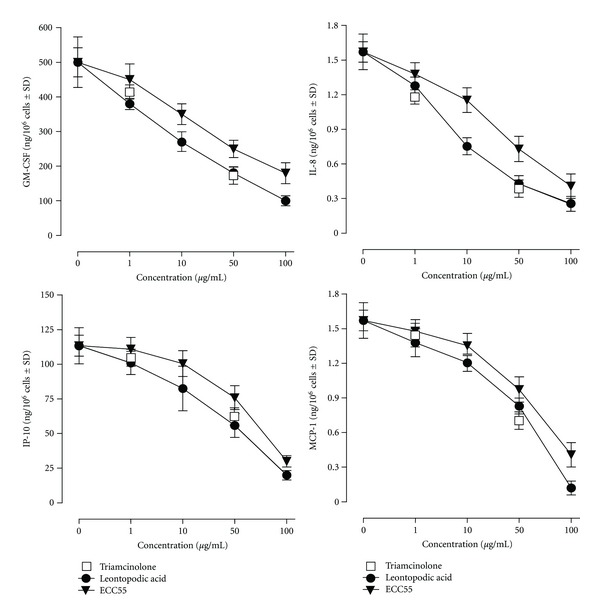
Dose-dependent effects of ECC55 and leontopodic acid on the chemokine and growth factor released from cultured human keratinocytes treated with proinflammatory combination of TNF*α* and IFN*γ* (T/I). Concentrations (ng/10^6^ cells) of granulocyte-macrophage colony-stimulating factor (GM-CSF), interleukin 8 (IL-8), interferon gamma-produced protein of 10 kDa (IP-10), and monocyte chemotactic protein-1 (MCP-1) were determined in the cellular supernatant at 24 h after keratinocyte challenge with T/I known to stimulate maximal inflammatory response. Keratinocytes were pretreated for 30 min with increasing concentrations (*μ*g/mL) of either ECC55 or pure commercial leontopodic acid. The corticosteroid triamcinolone was used as a positive control for effective inhibition of cytokine/chemokine release at the two concentrations indicated. Error bars in all figures correspond to S.D. Background release of IL-8 was 0.29 ± 0.04 ng/10^6^ cells, IP-10 was 0.001 ± 0.000 ng/10^6^ cells, GM-CSF was 0.07 ± 0.01 ng/10^6^ cells, and MCP-1 was 0.018 ± 0.02 ng/10^6^ cells. Spontaneous production of IP-10 and MCP-1 was not changed in the presence of either ECC55 or pure commercial LA, whereas basal GM-CSF and IL-8 levels were decreased by ECC55 and LA dose dependently.

Not surprisingly, T/I strongly activated inflammatory chemokines (IL-8, MCP-1, and IP-10) and growth factor (GM-CSF) release from PHK (Figure 3). Both ECC55 and LA dose dependently inhibited these inflammatory responses. Importantly, LA closely paralleled the activity of equal concentrations of the corticosteroid triamcinolone used as an effective inhibitor of cytokine/chemokine release in PHK and human skin (Figure 3). Concentrations of 50% inhibition (IC_50_) for GM-CSF were 23 *μ*g/mL for LA and 50 *μ*g/mL for ECC55, which reflects that exclusively LA could be responsible for the inhibitory action (calculated IC_50_ for LA within ECC55 is 28 *μ*g/mL). At the same time, IC_50_ for IP-10 inhibition was similar (51 *μ*g/mL and 72 *μ*g/mL for LA and ECC55, resp.). The same was observed for MCP-1 inhibition as well (51 *μ*g/mL and 66 *μ*g/mL for LA and ECC55, resp.). This allowed us to suggest that other major phenolic constituents of ECC55, such as chlorogenic and/or 3, 5 dicaffeoylquinic acids, could also exert inhibitory effects towards IP-10 and MCP-1 overexpression, as their anti-inflammatory activities towards epithelial cells have been reported previously [30, 31]. Regarding IL-8 inhibition, ECC55 was much less effective than pure LA (IC_50_ 9 *μ*g/mL for LA versus 43 *μ*g/mL for ECC55). The low ECC55 inhibitory action could be explained by the possible presence of IL-8-inducing plant polyphenols in the extract. For example, we repeatedly observed *de novo* synthesis of IL-8 in PHK treated with resveratrol or its glycosylated derivative polydatin [18, 28]. The identification of IL-8 promoting phenols in the Edelweiss extracts should be performed.

### 3.5. Extract of *Leontopodium alpinum* Callus Cell Cultures (ECC55) Reverses Keratinocyte Responses to Trichostatin A: An Inhibitor of Sirtuins

**Figure 4:**
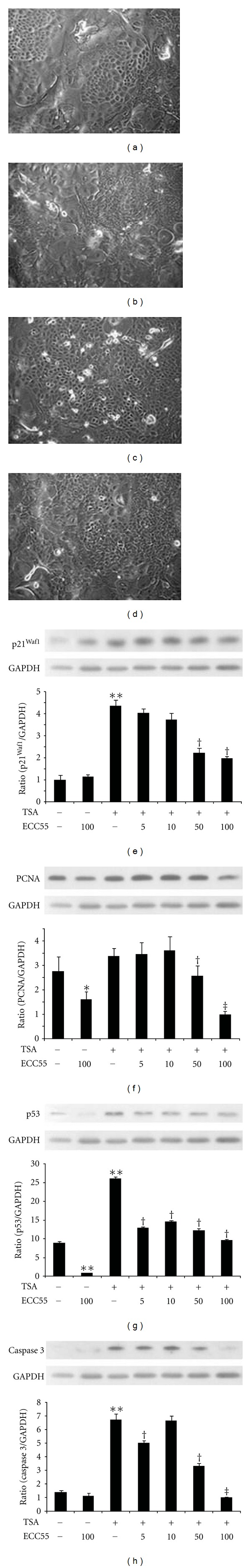
Effects of concentrated ethanol extract of Edelweiss callus cultures (ECC55) on Trichostatin A-induced cellular senescence and molecular markers of apoptosis, cell cycle, and proliferation. (a)–(d). Phase contrast microphotographs of cultivated human keratinocytes: untreated control (a), treated with Trichostatin A alone (b), treated with ECC55 alone (c), and treated with a combination of Trichostatin A and ECC55 (d). (e) Western blots and their densitometry showing p21 expression upon treatment with 100 *μ*g/mL ECC55 alone, Trichostatin A (TSA) alone, or TSA combined with increasing concentrations of ECC55 (5–100 *μ*g/mL). (f) The same for proliferation-connected nuclear antigen (PCNA). (g) The same for p53 and (h) for caspase 3. Error bars in all figures correspond to S.D. *P < 0.05 and **P < 0.01 versus untreated control; ^†^ *P* < 0.01 and ^‡^ *P* < 0.001 versus TSA-treated keratinocytes.

Histone deacetylase inhibitors, such as trichostatin A (TSA), induce a senescence phenotype in cultivated human fibroblasts [32] and in keratinocytes, [33] and Figure 4 (panel b versus panel a). The pretreatment of PHK with ECC55 did not affect normal keratinocyte morphology (Figure 4, panel c versus panel a) and prevented cells from TSA-induced senescence and reduced proliferation (Figure 4, panel d versus panel b). More profound examination of ECC55 effects towards molecular markers, expression of which was affected by TSA [33], showed a dose-dependent decrease of TSA-induced p21^Waf1^, a cell-cycle regulator of G1 phase (Figure 4, panel e). The expression of the p21^Waf1^ protein is controlled by the tumor suppressor protein p53, which mediates the p53-dependent cell cycle arrest in G1 phase in response to stressful conditions [34]. The p21^Waf1^ protein is cleaved by caspase 3 playing a crucial role in apoptosis following the caspase activation. Accordingly, TSA induced overexpression of both apoptotic markers, p53 and caspase 3 in PHK. Expression of both proteins was significantly reduced by ECC55 before treatment (Figure 4, panels g and h). Moreover, EC55 itself at the concentration of 100 *μ*g/mL inhibited spontaneous p53 expression. In addition to growth arrest, p21 regulates cellular senescence in PHK and interacts with proliferating cell nuclear antigen (PCNA) [33]. Of importance, the proliferative marker PCNA was slightly increased upon action of 5 and 10 *μ*g/mL of ECC55, although at 100 *μ*g/mL, the extract significantly suppressed both spontaneous and TSA-connected PHK proliferation (Figure 4, panel f). On the grounds of these results, we concluded that ECC55 dose dependently opposed Trichostatin A-induced sirtuin-connected cellular events leading to keratinocyte senescence and apoptotic death. Recently, the key role of sirtuin activation in cell/organism aging, apoptosis, and inflammation has been extensively discussed [32, 33, 35, 36]. Pharmacological sirtuin inhibitors, such as Trichostatin A, have been under investigation as targeted apoptosis-inducing anticancer drugs [37]. On contrast, sirtuin activators, such as resveratrol and several other plant-derived polyphenols, have been studied as anti-inflammatory remedies, first of all to combat chronic colitis and attenuate sirtuin-connected inflammation in the intestinal epithelium [38–40]. In our hands, ECC55 showed remarkable sirtuin activating properties, which assumes its possible anti-inflammatory action through this molecular pathway.

### 3.6. Extract of *Leontopodium alpinum* Callus Cell Cultures ECC55 Selectively Inhibits LPS-Induced IL-6 and VCAM-1 Gene Expression and LDLox-Induced VCAM-1 Gene Expression in Cultured Human Endothelial Cells

**Figure 5:**
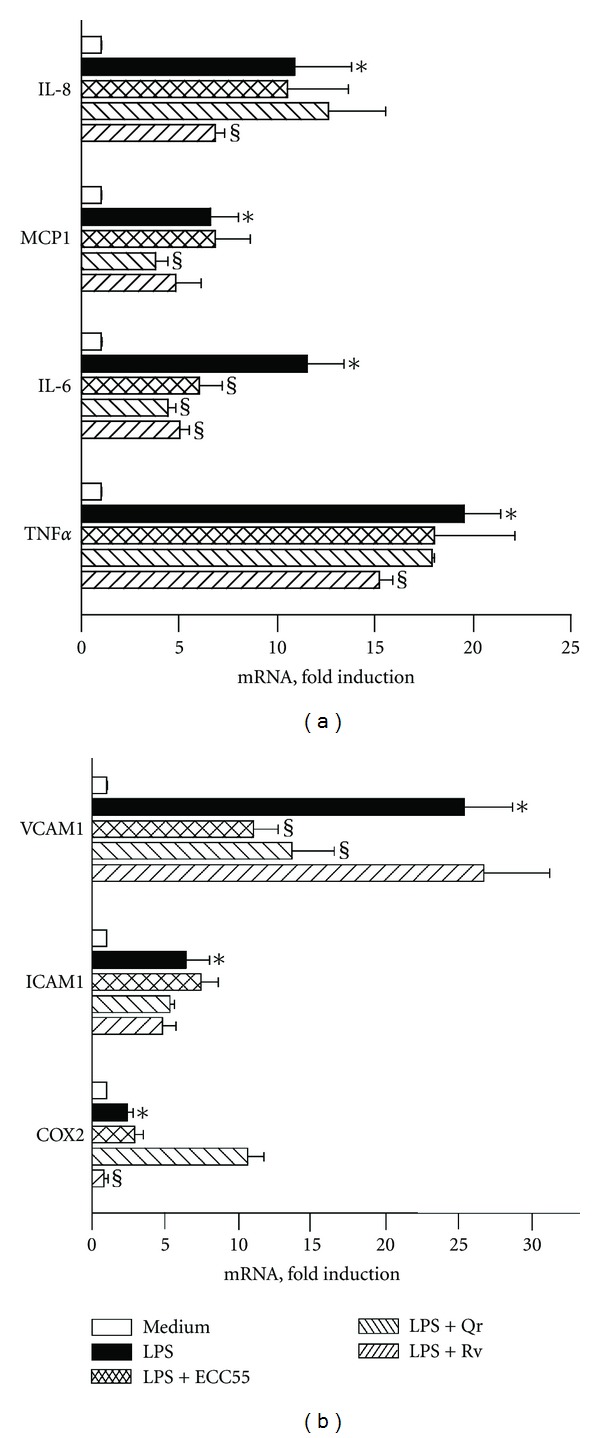
Effects of concentrated ethanol extract of Edelweiss callus cultures (ECC55) and reference anti-inflammatory plant polyphenols on inflammatory gene expression in human vascular endothelial cells (HUVECs) challenged with bacterial lipopolysaccharide (LPS). Abbreviations: Qr = quercetin, 25 *μ*g/mL; Rv = resveratrol, 25 *μ*g/mL; IL-8 = interleukin 8; MCP-1 = monocyte chemotactic protein-1; IL-6 = interleukin 6; TNF*α* = tumor necrosis factor alpha; VCAM1 = vascular cellular adhesion molecule-1; ICAM1 = intercellular adhesion molecule-1; COX2 = cyclooxygenase 2. Error bars in all figures correspond to S.D. *P < 0.01 versus untreated control; ^§^ *P* < 0.01 versus LPS-treated keratinocytes.

**Figure 6:**
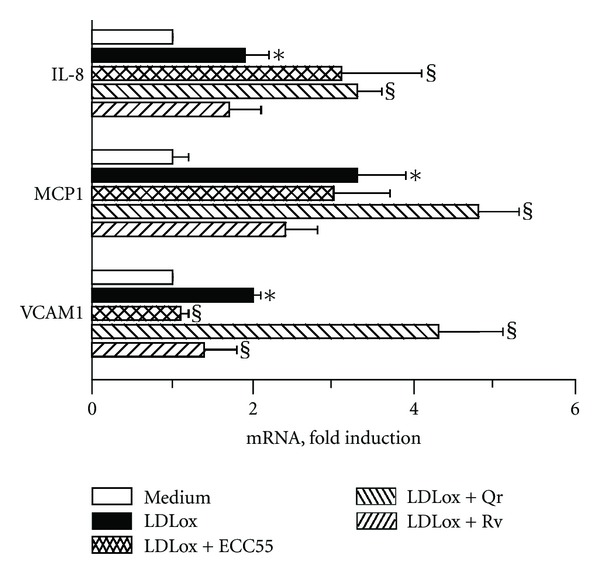
Effects of concentrated ethanol extract of Edelweiss callus cultures (ECC55) and reference anti-inflammatory plant polyphenols on inflammatory gene expression in human umbilical vein endothelial cells (HUVECs) challenged with oxidized human low-density lipoprotein (LDLox). Abbreviations are as in Figure 5. Error bars in all figures correspond to S.D. *P < 0.01 versus untreated keratinocytes; ^§^ *P* < 0.05 versus LDLox-treated keratinocytes.

Cellular adhesion molecules (CAMs), such as vascular cell adhesion molecule-1 (VCAM-1), play a key role in initiating inflammatory responses of the vascular system [41]. Their expression in endothelial cells exposed to inflammatory stimuli causes accumulation of activated polymorphonuclear leukocytes and monocytes, leukocyte sticking, rolling, and penetration through the endothelial layer. As a result, vascular tissue damage by leukocyte-derived inflammatory mediators occurs followed by accelerated proliferation of vascular cells. In our experimental setting, we stimulated inflammatory responses in HUVEC cells by incubating them with classical triggers: bacterial lipopolysaccharide (LPS), a crucial proinflammatory trigger characteristic for bacterial infections, and human oxidized low-density lipoprotein (LDLox), a trigger of atherogenic inflammation. These two stimuli induced differential patterns of inflammatory cytokines, growth factors, adhesion molecules, and proinflammatory enzymes (Figures 5 and 6), as it has been reported previously [20]. While LPS significantly induced genes encoding chemokines for polymorphonuclear leukocytes (*IL-8*) and monocytes (*MCP-1*), two early proinflammatory cytokines (*TNF*α** and *IL-6*), both CAMs expressed on endothelial cells (intercellular adhesion molecule (*ICAM-1)* and *VCAM-1*), as well as cyclooxygenase-2 (Figure 5), oxLDL induced *IL-8*, *MCP-1*, and *VCAM-1* exclusively (Figure 6). Of particular interest, ECC55 selectively and remarkably reduced *VCAM-1* gene expression in response to both triggers. None of the reference anti-inflammatory plant polyphenols studied (Qr and Rv) possessed such a selectivity, although all of them (ECC55, Qr, and Rv) significantly inhibited LPS-induced *IL-6* transcript (Figure 5). The molecular mechanism of VCAM-1 inhibition by Edelweiss extract deserves further profound investigation in order to provide a solid basis for its promising development as a targeted antivascular anti-inflammatory drug.

## 4. Conclusions

Concentrated Ethanol Extract of *Leontopodium alpinum* callus cell cultures containing phenolic fraction, 55% of which were leontopodic acids, possessed remarkable anti-inflammatory properties at both transcriptional and translational levels in the cultured human skin keratinocytes and endothelial cells, thus confirming the ethnopharmacological use of the parental herb. The extract inhibited proinflammatory pathways dose dependently, and the effects were cell- and proinflammatory trigger-specific. Interestingly, the *Leontopodium alpinum* extract opposed the sirtuin inhibitor Trichostatin A, thus, possibly, activating sirtuin-associated intrinsic anti-inflammatory mechanisms. Biotechnologically produced *Leontopodium alpinum* cell cultures could be a valuable and biodiversity protective source of the natural anti-inflammatory substances feasible for chronic inflammatory skin diseases and bacterial and atherogenic inflammation.

## Figures and Tables

**Table 1:** Quantification of phenolic extracts of aerial parts of Edelweiss plant, *Leontopodium alpinum* callus cultures, and concentrated extract enriched in leontopodic acids (ECC55).

Phenolics	Capitula of Edelweiss*(%) [12]	*Leontopodium alpinum* callus cultures (%)	ECC55 (%)
Chlorogenic acid	0.5	1.2	24
3,5-Dicaffeoylquinic acid	1.0	0.8	21
Leontopodic acid B	0.5	1.0	14
Leontopodic acid	3.0	2.2	41
Luteoline	Traces	—	
Apigenine	Traces	—	
Isoquercetin	Traces	—	
Luteolin-7,4′-di-O-*β*-D-glucoside	0.1	—	
Luteolin-4′-O-*β*-D-glucoside	0.3	—	
Luteolin-7-O-*β*-D-glucoside	Traces	—	
Luteolin-3′-O-*β*-D-glucoside	0.1	—	
Apigenin-7-O-*β*-D-glucoside	Traces	—	
Chrysoeriol-7-O-*β*-D-glucoside	Traces	—	
6-Hydroxy-luteolin-7-O-*β*-D-glucoside		—	
Pentahydroxyflavone	Traces	1.5	

*Content of chlorogenic acid, 3,5-dicaffeoylquinic acid, leontopodic acid B, and leontopodic acid was different in the stems, stem leaves, capitula, in-florescences leaves, and leaves of the basal rosette [12].
